# Spinal Muscular Atrophy with Progressive Myoclonic Epilepsy (SMA-PME): three new cases and review of the mutational spectrum

**DOI:** 10.1186/s13052-023-01474-z

**Published:** 2023-06-06

**Authors:** Ali Najafi, Behnoosh Tasharrofi, Farshid Zandsalimi, Maryam Rasulinezhad, Masood Ghahvechi Akbari, Gholamreza Zamani, Mahmoud Reza Ashrafi, Morteza Heidari

**Affiliations:** 1 Department of Medical Genetics, School of Medicine, Tehran University of Medical Sciences, Tehran, Iran; 2 Department of Molecular Medicine, School of Advanced Technologies in Medicine, Tehran University of Medical Sciences, Tehran, Iran; 3 Pediatric Neurology Department, Iran University of Medical Sciences, Tehran, Iran; 4 Physical Medicine and Rehabilitation Department, Children’s Medical Center, Tehran University of Medical Sciences, Tehran, Iran; 5 Department of Pediatrics, Division of Pediatric Neurology, Children’s Medical Center, Pediatrics Center of Excellence, Tehran University of Medical Sciences, Tehran, Iran

**Keywords:** Acid ceramidase, SMA-PME, *ASAH1* gene, Whole exome sequencing

## Abstract

**Background:**

Spinal muscular atrophy (SMA) could be classified as 5q and non-5q, based on the chromosomal location of causative genes. A rare form of non-5q SMA is an autosomal-recessive condition called spinal muscular atrophy with progressive myoclonic epilepsy (SMA-PME), phenotypically characterized by myoclonic and generalized seizures with progressive neurological deterioration. SMA-PME is a clinically heterogeneous disorder that arises from biallelic pathogenic variants in *ASAH1* gene.

**Methods:**

Following clinical and primary laboratory assessments, whole-exome sequencing was performed to detect the disease-causing variants in three cases of SMA-PME from different families. Also, Multiplex ligation-dependent probe amplification (MLPA) was employed for determining the copy numbers of *SMN1* and *SMN2* genes to rule out 5q SMA.

**Results:**

Exome sequencing revealed two different homozygous missense mutations (c.109 C > A [p.Pro37Thr] or c.125 C > T [p.Thr42Met]) in exon 2 of the *ASAH1* gene in the affected members of the families. Sanger sequencing of the other family members showed the expected heterozygous carriers. In addition, no clinically relevant variant was identified in patients by MLPA.

**Conclusion:**

This study describes two different *ASAH1* mutations and the clinical picture of 3 SMA-PME patients. In addition, previously reported mutations have been reviewed. This study could help to fortify the database of this rare disease with more clinical and genomic data.

## Background

Spinal muscular atrophy (SMA) is a neuromuscular disease affecting nerves and skeletal muscles by progressive degeneration of motor neurons in the spinal cord and brain stem. It includes a heterogeneous group of disorders, which may be classified into two subtypes each depending on its own genes: *SMN1* and *SMN2* genes are involved in about 95% of cases and define the so called “5q SMA” group. The other one is called “non-5q SMA”, and is caused by mutations in 20 genes including *ASAH1* [1].

*ASAH1* is a relatively small gene containing 14 exons. It is located on 8p22 chromosome and encodes the acid ceramidase enzyme (aCDase) which breaks down ceramide into sphingosine and free fatty acid. Its alternative splicing results in multiple transcripts, among which at least one encodes a proteolytically processed preproprotein. This generates a protein, consisting of a non-glycosylated alpha and a glycosylated beta subunits, which is cleaved to the mature lysosomal enzyme [2, 3]. Since *ASAH1* is ubiquitously expressed, mutations in this gene result in lysosomal accumulation of ceramides in various tissues which eventually causes the phenotypical manifestations [4].

So far, 107 pathogenic and 49 likely pathogenic variants have been reported for this gene in ClinVar (https://www.ncbi.nlm.nih.gov/clinvar/), among which the most frequent are missense. These mutations lead to aCDase deficiency mainly causing Farber disease and rarely the phenotype of spinal muscular atrophy with progressive myoclonic epilepsy (SMA-PME, OMIM 159950). Farber is usually a neonatal or early infantile disease characterized by a hoarse voice or a weak cry, small lumps of fat under the skin and in other tissues (lipogranulomas), and painful contractures of joints [5]. Affected individuals may also have difficulty in breathing, hepatosplenomegaly, and developmental delay [6]. SMA-PME was first described by Jankovic and Rivera in 1978 but the disease phenotype was linked to the *ASAH1* gene in 2012 [7]. It is an autosomal recessive disorder representing a heterogeneous group of epilepsies, usually starting within 2–6 years of age with muscle atrophy, difficulty in walking and tremors, and later developing into myoclonic epilepsy usually during late childhood. Since the first symptoms of SMA-PME may overlap with those of“5q SMA”, it is usually necessary to carry out genetic testing for diagnosis confirmation and to rule out 5q SMA disease [8]. No successful treatment has been reported until now, and patients are managed by symptomatic multidisciplinary treatments [9].

Since only 37 genetically confirmed SMA-PME patients have been reported to date [6, 8, 10–17], most of which show the p.T42M mutation, new cases and mutations can significantly contribute to a better understanding of the disease. Herein, we present 3 cases of SMA-PME with homozygous missense mutations, and briefly compare them with previously reported missense mutations of *ASAH1* causing SMA-PME.

## Materials and methods

### Patients

#### Family A

A 6-year-old boy with complaints of slowly progressive muscle weakness especially in lower limb muscles and polyminimyoclonic movements was referred to the SMA Clinic, Children’s Medical Center, Tehran, Iran. He was born by a non-consanguineous couple and preterm delivery (33 weeks of gestation). His mother had 2 spontaneous abortions and there was history of intellectual disability and behavioral disorders in the pedigree. He had normal development until his 3rd year of life when his motor regression was noted by difficulty in running, climbing stairs, and standing up. At the same time, he started manifesting jerking movements without losing consciousness. The muscle weakness was mildly progressive and in the last visit in May 2022 when he was 9, the proximal upper limb muscles force, evaluated according to the Medical Research Council (MRC), was 4/5 and the proximal lower limb muscles scored 3/5. His Gower sign was positive, and he could not rise from the ground without help. Progressive weakness of neck flexor and extensor muscles occurred since the age of 7. He also had at resting tremor, fasciculations, mild lordosis, bilateral ocular telangiectasia, and seasonal allergy requiring inhalation medication. Furthermore, there were no cognitive problems or speech disorders. Also, plasma creatine kinase (CK), lactate dehydrogenase (LDH), liver enzymes and the function of auditory nerves, checked by ABR test, are currently normal. Brain MRI showed no lesions or remarkable signal changes. Electrodiagnostic (EDX) evaluations were performed by Nihon-Kohden electromyograph. All Nerve Conduction Studies (NCSs) were normal. Needle Electromyography (EMG) demonstrated neurogenic changes with high amplitude and long-duration motor unit action potentials (MUAPs), associated with reduced recruitment and spontaneous potentials such as fibrillation (Fib), positive sharp waves (PSW), and fasciculation (Fasc). These findings confirmed a chronic denervation process with ongoing regeneration that was compatible with the category of anterior horn cell diseases. Electroencephalography (EEG) showed abnormal results due to slow activity besides some polyspike-wave activity, hence, Depakine administration was started. He has not experienced to date either respiratory problems or swallowing difficulties. 

#### Family B

**Fig. 1:**
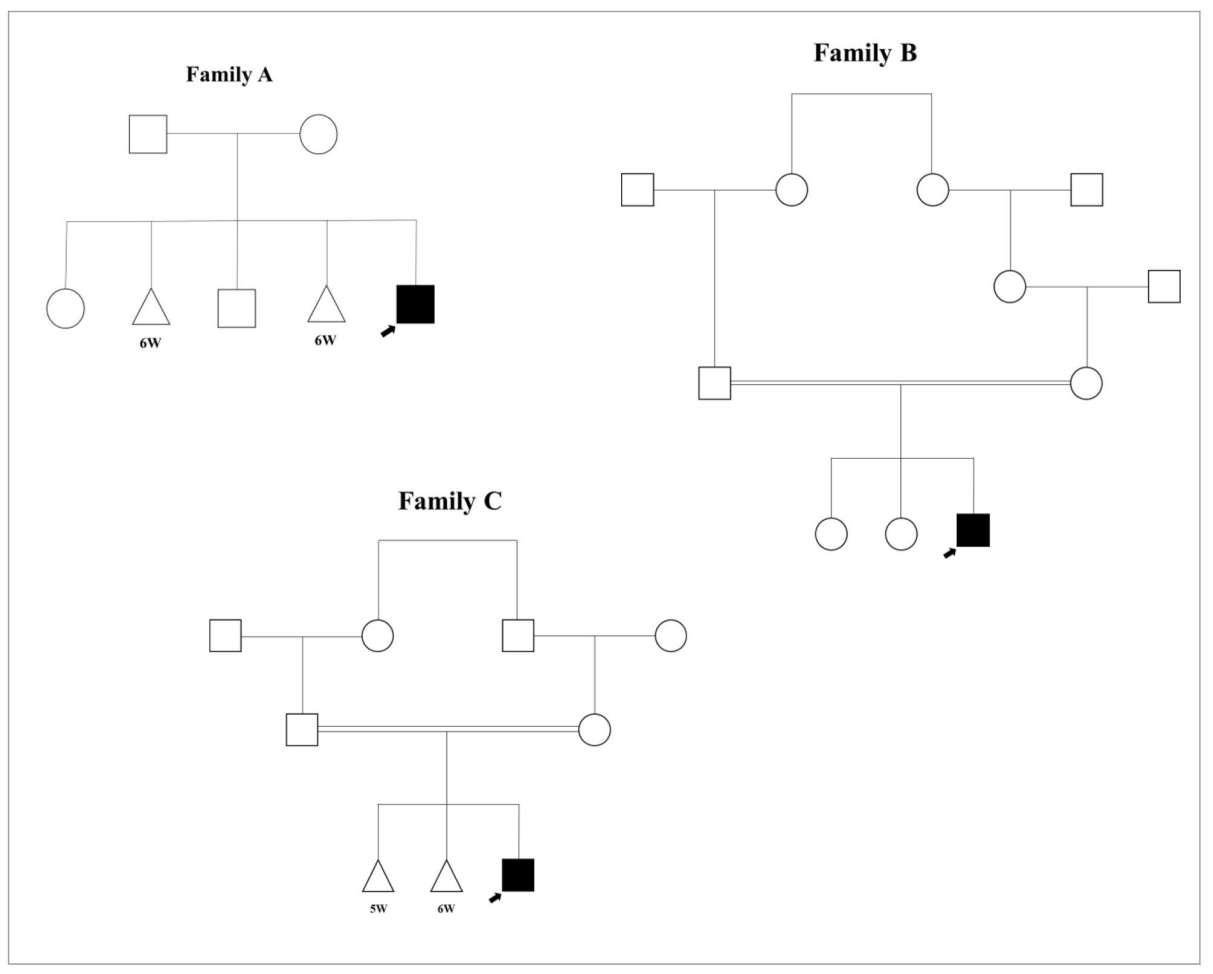
Pedigree of the 3 Iranian families affected by SMA-PME. Family A, with an affected boy with c.109 C > A variant in *ASAH1* gene. Families B and C, the 2 consanguineous families with common c.125 C > T variant in *ASAH1* gene. The affected probands are indicated by black arrow. Circles indicate female, squares indicate male, and triangles indicate abortion. Consanguinity is represented by parallel lines in families B and C

The proband is a 7-year-old boy with normal neurodevelopmental milestones. He was born by a consanguineous marriage, and there was no history of disease in the pedigree (Fig. 1). Since the age of 3 years he has started to develop lower limb muscular weakness presenting with frequent falling. His disease continued with the problem of standing and climbing stairs. Paraclinical investigations including CK and LDH were within normal limits. He has never had seizures or respiratory problems and his symptoms have slightly progressed.

#### Family C

The proband is a 7-year-old boy with difficulties in standing, climbing stairs and putting her feet outwards since he was 4. His parents were first cousins, and the mother had two miscarriages. His disease has not quickly progressed to date; indeed, seizures as well as respiratory, swallowing and speech problems are not presently observed.

### Multiplex ligation-dependent probe amplification (MLPA)

DNA was extracted from peripheral blood and detection of *SMN1* and *SMN2* gene copy numbers was done using a commercially available Salsa MLPA kit P021 (MRC-Holland), according to the manufacturers’ instructions (http://www.mrc-holland.com). PCR products were analyzed on the ABI 3130 Genetic Analyzer (Applied Biosystems), and data were processed by Coffalyzer software (MRC Holland).

### Whole exome sequencing and variant confirmation

Exons of the patients’ DNA were enriched using Agilent SureSelect V7 and paired-end sequencing was performed by Illumina NovaSeq sequencer (NovaSeq 6000, **Montréal**, Canada). The variants were annotated and filtered using the American College of Medical Genetics (ACMG) guidelines for the interpretation of sequence variants [18]. This included comparison against the gnomAD population catalog of variants in 123,136 exomes, the Iranome catalog of genomic variants in the Iranian population with 800 healthy individuals, the 1000 Genomes project Consortium’s publication of 2500 genomes, the NCBI ClinVar database of clinical assertations on variant’s pathogenicity and multiple lines of computational evidence on conservation and functional impact. Here, the used *in silico* prediction tools were Sorting Intolerant from Tolerant (SIFT), Polymorphism Phenotyping version 2 (PolyPhen-2), MutationTaster (http://www.mutationtaster.org), Protein Variation Effect Analyzer (PROVEAN), Functional Analysis through Hidden Markov Models (FATHMM), Combined Annotation Dependent Depletion (CADD) and DANN (a deep learning approach for annotating the pathogenicity of genetic variants) [19]. In order to prioritize genes responsible for neuromuscular diseases, OMIM (Online Mendelian Inheritance In Man) and PubMed were used.

To confirm the variants, a specific set of primers was designed for amplification of exon 2 of the *ASAH1* gene (NM_177924.5) and Polymerase Chain Reactions (PCR) were performed in 35 cycles.

Exon 2 of the *ASAH1* gene (NM_177924.5), including the variants of interest, was sequenced by Big Dye Terminators (Applied Biosystems 3130 Genetic Analyzer, Foster City, CA). This step was performed for patients in addition to their parents. (Sequences available upon request).

### Analysis of acid ceramidase structural stability and pathogenicity of all known missense mutations of ***ASAH1***

One of the common methods for evaluating the effect of a missense variant on protein stability is to calculate Gibbs free energy changes (ΔΔG). We used FoldX plugin (version 4) on YASARA program (version 22.8.21) for this purpose [20]. First, human acid ceramidase (aCDase) structure was obtained from Protein Data Bank (https://www.rcsb.org/) with PDB code 5U7Z. Then, energy optimization was performed with FoldX. The stability of this structure was assessed by calculating its free energy (ΔG_wt_). All reported mutations of *ASAH1* causing SMA-PME were introduced separately to the structure of aCDase. Energy optimization and calculation of the free energy (ΔG_mt_) was performed. Finally, ΔG changes were calculated with the following formula:

ΔΔG = ΔG_mt_ –ΔG_wt_.

Also, to evaluate the deleteriousness and disease causing potential of the mutations, SIFT [21], PolyPhen2 [22], Mutation Taster [23] and CADD Phred score [24] were calculated for all the missense mutations.

## Results

### MLPA result for ***SMN1*** and ***SMN2***

Results of MLPA for all the 3 patients showed that none of them harbored a homozygous deletion in *SMN1*. Summary of the test results is shown in Table 1.

**Table 1:** *SMN1* and *SMN2* copy numbers. None of the patients had homozygous deletion of *SMN1* or *SMN2*.

	*SMN1* copy number	*SMN2* copy number
**Patient 1**	2	1
**Patient 2**	2	2
**Patient 3**	1	2

### Whole exome sequencing and variant confirmation results

Whole exome sequencing led to the identification of a homozygous variant [NM_177924.5: c.109 C > A; (p.Pro37Thr)] for family A in the *ASAH1* gene. This variant was absent from gnomAD, the 1000 Genomes Consortium (http://www.internationalgenome.org/home), and Iranome databases. The mutation is predicted to be damaging by protein prediction algorithms. Finally, the candidate variant was classified as Likely Pathogenic based on the American College of Medical Genetics and Genomics (ACMG) guidelines. Furthermore, c.125 C > T (p.Thr42Met) variant was detected in homozygous status for families B and C. This variant has very low frequency in population databases and predicted to be damaging by *in silico* computational analysis. It was previously reported as a pathogenic variant in SMA-PME patients (Table 2).

**Table 2:** Features of the two variants found in the *ASAH1* gene

Family	Gene	Variant	Exon	Allele frequency	Zygosity	Type and classification
**A**	*ASAH1*	NM_177924.5 c.109 C > A p.Pro37Thr	2/14	gnomAD: N/A 1KGP^*^: N/A Iranome: N/A	Homozygous	Missense, Likely Pathogenic
**B, C**	*ASAH1*	NM_177924.5 c.125 C > T p.Thr42Met	2/14	gnomAD: 3 1KGP^*^: N/A Iranome: N/A	Homozygous	Missense, Pathogenic

* 1000 Genome Project

**Fig. 2:**
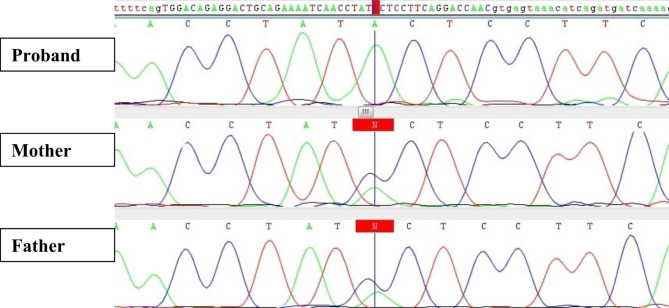
Sanger sequencing chromatogram of family A. The proband is homozygous for the c.109 C > A variant in *ASAH1* gene. His parents are both carrier for this variant

Based on the results of Sanger sequencing in the families, the c.109 C > A and c.125 C > T variants in *ASAH1* gene were confirmed to be in homozygous state in the probands and in heterozygous state in their parents (Fig. 2). As expected, none of the family members except the proband had any signs and symptoms of SMA-PME, due to its autosomal recessive inheritance.

**Investigation of aCDase thermal stability and the pathogenicity of missense mutations of*ASAH1***.

**Fig. 3:**
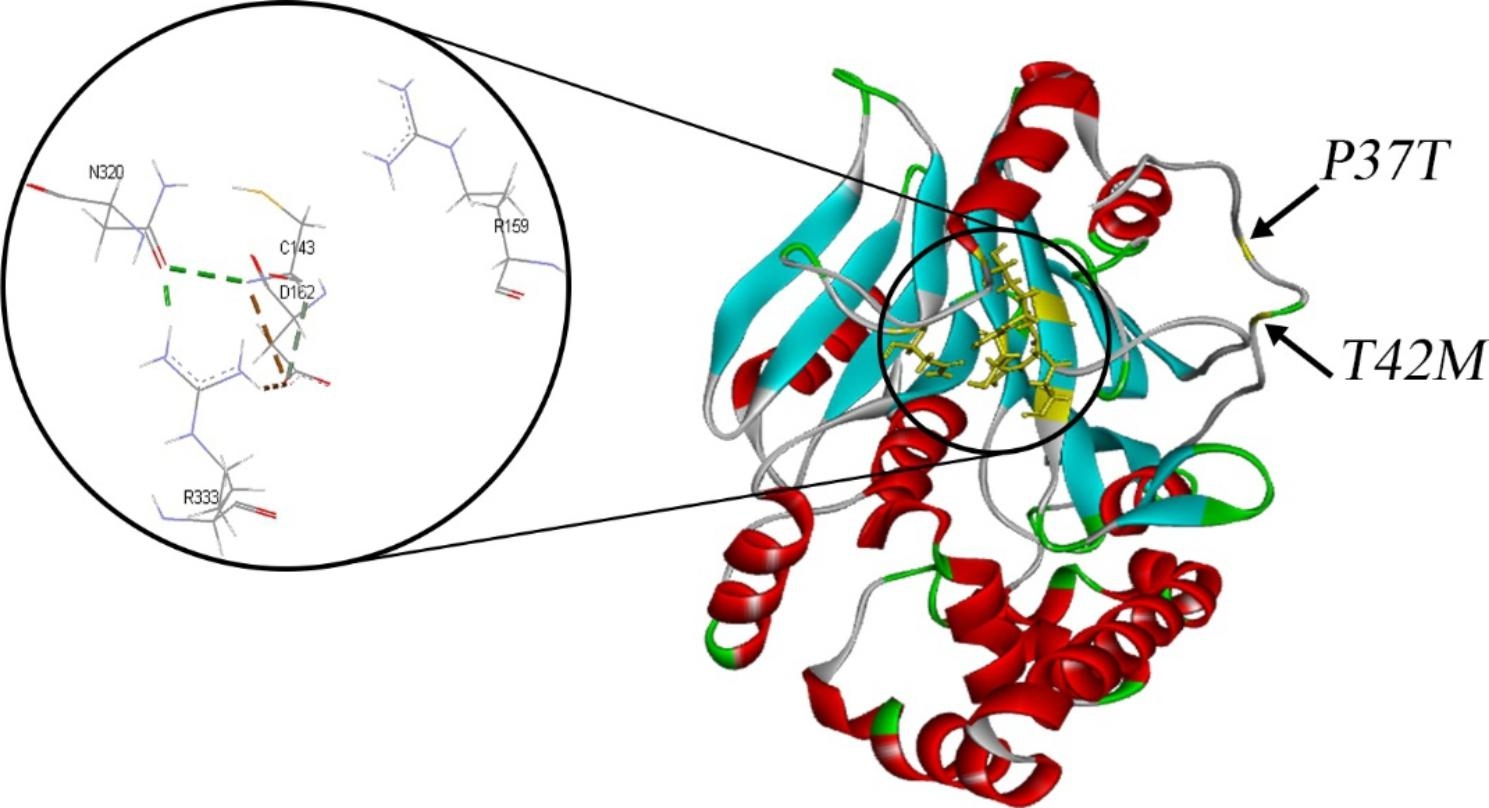
The structure of aCDase. The position of amino acids changed in our patients are indicated by arrows. Active site of the enzyme is magnified in circle. Dashes represent the possible interaction between amino acids of the active site

The free energy change (ΔΔG) of the activated aCDase was calculated with FoldX plug-in on YASARA software (version 20.12.24) for all reported mutations of *ASAH1*-related SMA-PME cases (Table 3). The ΔΔG for the p.Pro37Thr variant was 2.848 which favors protein destabilization. By contrast, the ΔΔG of p.Thr42Met mutation was − 0.939 and indicates that it can be considered as a stabilizing mutation. The position of these 2 mutations of our patients in relation to the active site of aCDase are depicted in Fig. 3. The range of ΔG change in all the included mutations were between − 0.939 and 6.262.

**Table 3:** Comparison of all missense variants of the *ASAH1* gene (NM_177924.5) reported to cause SMA-PME.

cDNA change	Amino acid change	Protein chain	Thermal stability changes (Kcal/mol)	SIFT & PolyPhen2	Mutation Taster	CADD PHRED score	Frequency in gnomAD (Aggregated)
c.109 C > A	P37T	α-subunit	2.848	Deleterious &Probably Damaging	Disease causing	27.1	0
c.125 C > T	T42M		-0.939	Deleterious & Probably Damaging	Disease causing	32	3
c.124 A > G	T42A		-0.133	Tolerated &Benign	Disease causing	24	4
c.410 A > G	Y137C		4.601	Deleterious &Probably Damaging	Disease causing	26.6	6
c.456 A > C	K152N	β-subunit	− 0.585	Tolerated &Benign	Disease causing	22.5	16
c.518 A > T	N173I		− 0.148	Tolerated &Probably Damaging	Disease causing	24.9	0
c.536 C > T	T179I		6.262	Deleterious &Probably Damaging	Disease causing	26.3	2
c.556 A > G	T186A		1.709	Deleterious & Benign	polymorphism	22.5	2
c.620 A > T	Y207F		0.274	Tolerated &Probably Damaging	Disease causing	24.3	105
c.1178T > C	I393T		0.493	Tolerated &Benign	Disease causing	20.8	0

Prediction of the effect of the mutations were also assessed with SIFT, PolyPhen2, Mutation Taster and CADD Phred score [24, 25]. These prediction tools showed that the 2 variants of our patients are deleterious and disease causing with relatively high scores in CADD tool. Results of these prediction tools are summarized in Table 3.

## Discussion

SMA is one of the most common neuromuscular disorders with pediatric lethality [26]. In about 95% of cases, SMA is caused by deletions in exons 7 and 8 of SMN1 gene. This gene is on chromosome 5 (5q13), so, the relevant SMA is called 5q SMA. Other less prevalent types of SMA which are not due to mutations in this gene, are called non-5q SMA. Mutations in 20 genes including *ASAH1* have been identified to be responsible for “non-5q SMA”, about half of *ASAH1*-related mutations are T42A and T42M occurring in exon 2 [14]. The number of SMA-PME and SMA-PME like patients reported up to 2018 were respectively 23 and 20, accounting for a total of 43 cases [14]. Moreover, so far, 58 cases of SMA-PME have been reported, 37 of which have been confirmed by genetic testing. In this study, we investigated 3 patients from unrelated families by clinical examination and genetic testing carrying two pathogenic or likely pathogenic variants in the *ASAH1* gene. Mutations in *ASAH1* cause Farber disease and SMA with progressive myoclonic epilepsy (SMA-PME). Farber disease is severe, showing infancy onset and a median survival period of about 3 years [27]. Its classic features include the triad of subcutaneous nodules, joint swellings/arthritis and hoarse or weak voice [14]. Typically, activity of the aCDase is more markedly reduced in Farber disease than SMA-PME [14]. Zielonka et al. showed that a higher residual activity of aCDase is associated with later onset and longer survival of Farber patients [27]. A similar pattern seems to be true also for SMA-PME which is an ultrarare (Prevalence: **<**1/1 000 000**)** [28–30] childhood neurological condition leading to muscle weakness and atrophy. It also manifests seizures and uncontrollable myoclonic epilepsy [8]. The main cause of death in SMA-PME patients is respiratory insufficiency, usually occurring 5 to 15 years after the onset of the disease. All our patients are alive.

One of the variants here reported is a missense mutation, carried by the patient belonging to family A, who showed progressive muscle weakness, seizures, fasciculation, and scleral telangiectasia. Previous studies indicate all these symptoms related to SMA-PME patients except for the last one [4, 14, 31]. The present finding of ocular telangiectasia may expand the clinical phenotype of the disease. T42M is the variant harbored by the other 2 patients of our study. Since various mutations diversly affect structure and function of the protein, a number of studies indicate that the substitution of threonine by methionine at position 42 of the encoded protein causes a milder effect on the final product so that acid ceramidase activity is partially preserved reaching 30% [32]. As expected, the present patients with T42M mutation have started to show muscular weakness with a slowly progressive pattern, reflecting their likely less severe type of mutation.

Previously one patient has been reported from Iran with p.T42M mutation in *ASAH1* and classic symptoms of SMA-PME [12]. Here, we describe 3 new cases. Two of our patients are born by consanguineous parents, but the patient related to family A, whose parents belong to a small city in west of Iran, was born by a non-consanguineous marriage. Regarding the latter case, it should be noted that ancient consanguinity, especially in such a small town cannot be excluded [33]. The average rate of consanguineous marriage in Iran is 37.4% which puts it among the highest rates in the world [34, 35]. Therefore the rate of recessively inherited disorders are expected to be higher than western countries with very low rates of consanguineous marriages [36]. In accordance with previous studies showing higher rates of congenital abnormalities including inborn errors of metabolism in presence of parental consanguinity [37], most of the reported SMA-PME cases as well as 2/3 of our patients are born by consanguineous parents [6, 31, 35].

For the confirmation of SMA-PME it is worth to assess aCDase enzyme activity. However, one limitation of our study was unavailability of this test in Iran. So, we have confirmed the SMA-PME cases based on clinical, primary laboratory and subsequent genetic evaluations. However, we showed that the aCDase structural stability could be deteriorated with the destabilizing (ΔΔG > 0) p.Pro37Thr mutation. The other variant (p.Thr42Met) was a slightly stabilizing (ΔΔG < 0) mutation. Although the structural prediction scheme classified the current mutation as a stabilizing change, it is noteworthy to mention here that the thermodynamic stability of proteins is not the conclusive determining factor of mutation effects. Point mutations can contribute to the development of human diseases by disturbing protein-protein interaction (PPI) networks [38]. Such mutations, even located far from the active site, can also induce biophysical mechanisms that affect the affinity of substrate-enzyme interaction resulting in the malfunction of the enzyme [39].

*In silico* prediction for both of the substitutions with SIFT showed the deleteriousness of them. The PolyPhen2 analysis indicated these changes as ‘probably damaging’ and Mutation taster program predicted them as disease-causing. Also, they had relatively high CADD Phred scores. These bioinformatic analyses were in accordance with the causality of these two mutations in our patients. Moreover, these bioinformatic analyses were performed for all previously reported missense mutations of *ASAH1* known to cause SMA-PME (Table 3). While these prediction tools cannot fully substitute the functional assays for assessment of genetic variants, they can provide a broad insight into their pathogenicity.

## Conclusion

We performed MLPA and Whole Exome Sequencing (WES) for 3 patients affected with SMA-PME, and found a rare mutation (c.109 C > A; [p.Pro37Thr]) in 1 patient and a previously well-known mutation (c.125 C > T [p.Thr42Met]) in the other 2 patients. Both of them cause SMA-PME disease, due to deficiency of the aCDase enzyme. The patient with the rarer mutation in the *ASAH1* gene also manifested ocular telangiectasia, further expanding the clinical phenotype of SMA-PME.

Next-generation sequencing (NGS) is a robust genetic method for analyzing human genome, and WES as a more cost-effective version of NGS techniques, significantly accelerates the detection of disease-causing genetic variations [40, 41]. This powerful technique aids clinicians in the diagnostic process of genetic disorders with clinical and/ or genetic heterogeneity, and can help in family planning through genetic counselling about recurrence risk and primary prevention options including prenatal diagnosis (PND) or preimplantation genetic diagnosis (PGD) [42, 43]. Finally, it is noteworthy that NGS may help to formulate more accurate prognosis evaluation and individualized follow-up, based for each single case on the specific genomic profile [44].

## Data Availability

The datasets used and/or analyzed during the current study are available from the corresponding author on reasonable request.
